# Organizational Impact of Immunotherapies in Advanced Cancers in France

**DOI:** 10.1200/GO.23.00026

**Published:** 2023-08-18

**Authors:** Valentine Grumberg, Christos Chouaïd, Anne-Françoise Gaudin, Christophe Le Tourneau, Aurélien Marabelle, Isabelle Bongiovanni-Delarozière, François-Emery Cotté, Isabelle Borget

**Affiliations:** ^1^ Bristol Myers Squibb France, Rueil-Malmaison, France; ^2^ Oncostat, U1018, CESP, Inserm, Paris-Saclay University, “Ligue Contre le Cancer” Labeled Team, Villejuif, France; ^3^ Department of Chest Medicine, Créteil University Hospital, Créteil, France; ^4^ INSERM U955, UPEC, IMRB, Créteil, France; ^5^ Department of Drug Development and Innovation (D3i), Institut Curie, Paris, France; ^6^ INSERM U900 Research Unit, Paris-Saclay University, Saint-Cloud, France; ^7^ Drug Development Department (DITEP), INSERM U1015, Université Paris Saclay, Gustave Roussy, Villejuif, France; ^8^ Real-World Solutions, IQVIA France, Paris, France; ^9^ Department of Biostatistics and Epidemiology, Gustave Roussy, Paris-Saclay University, Villejuif, France

## Abstract

**PURPOSE:**

In 2020, the French National Authority for Health (*Haute Autorité de Santé*) published a methodologic guide called organizational impact (OI) cartography to define and structure assessment of the OI of health technologies. As immunotherapies are associated with extended survival and improved quality of life in advanced cancer, we aimed to identify OIs that immunotherapies had on health care systems and professionals. To our knowledge, we suggest the first implementation for OI assessment on the basis of the cartography.

**METHODS:**

A literature review was conducted, and interviews with health care professionals (HCPs) were performed to identify OIs of immunotherapies. They were asked if immunotherapies had OIs classified into three macrocriteria, namely, impact on the care process (six criteria), impact on capacities and skills required (six criteria), and impact on society (four criteria). If an OI was mentioned for a criterion, information on its impact (minor/moderate/major) and its timing was collected. We considered that an OI existed when 75% of HCPs mentioned an impact for a given criterion.

**RESULTS:**

Overall, 27 HCPs were interviewed. For 12 of 16 criteria, most HCPs mentioned an impact, whereas the literature identified impacts for 11 criteria. Four criteria (skills and transfer between HCPs, scheduling capabilities, and social relationship) had consensus among HCPs and a high impact; two criteria (rhythm or care duration, working/living conditions) showed consensus but a moderate impact; two criteria (funding and scheduling capabilities cross-structure) had a high impact but no consensus. For eight criteria (as environment or inequity), there was no consensus and moderate impact.

**CONCLUSION:**

The introduction of immunotherapies for advanced cancer has had an important OI in France, regarding capacities and skills. Further research using qualitative analysis of interviews will provide more information regarding OI.

## Teaser

An identification of organizational impacts related to immunotherapies in advanced cancer in France.

## INTRODUCTION

With 18.1 million new cases diagnosed and 9.6 million deaths per year, cancer is one of the major public health challenges in many countries.^1^ Cancer incidence will grow over time.^2^ At this time, more than 40% of patients with cancer are eligible for immune checkpoint inhibitor (ICI) treatment.^3^ These treatments have rapidly become the standard of care in many indications. ICIs double the proportion of patients achieving durable response compared with the previous standard of care (25% *v* 11%).^4^ Between 2014 and 2020, ICIs allowed us to gain 23,788 life years and 18,369 quality-adjusted life years compared with the previous standard of care at the population treated level in France.^5^ By improving survival, ICIs have also strongly modified the management of patients with advanced cancers and have had an important budget impact. To date, the overall organizational impact (OI) of this new therapeutic class for the health care system has not been investigated.

**CONTEXT**

**Key Objective**
To understand how to assess the organizational impact (OI) of health technologies on the basis of the cartography published by the Haute Autorité de Santé (HAS) and to identify OIs related to immunotherapies in advanced cancers.
**Knowledge Generated**
If immunotherapies have improved survival prognosis in multiple cancers, this therapeutic class has also had an OI on health care. They have increased treatment duration affecting resource utilization and had a major impact on the capabilities and skills required from health care professionals to implement the care process.
**Relevance**
To our knowledge, this is the first published work on OI in France using the cartography of the HAS. Health technology assessment should integrate OI analyses to understand overall consequences of innovative treatments. These analyses will help to optimize long-term health care organization.

In France, all new treatments that have received marketing authorization are evaluated by the Haute Autorité de Santé (HAS), the National Health Technology Agency (HTA), to determine price and reimbursement status. The assessment is composed of an evaluation of the actual benefit (AB) and the clinical added value (CAV) of the health technology by Transparency Committee (TC). An economic evaluation regarding the methodology of the cost-effectiveness analysis can be performed by the Economic and Public Health Committee (CEESP). The AB rating determines the degree of reimbursement, whereas the CAV and CEESP opinions are used for price negotiation. Until recently, an OI could be mentioned or claimed in the dossiers submitted to the HAS for the assessment of the AB, but no methodology on how to assess the potential OI was published.^6^ For this reason, this aspect has rarely been documented.

In December 2020, the HAS published an OI cartography (OIC) for health technology assessments.^7^ In this document, the HAS sought to clarify the aspects associated with the OI of health technologies. An OI is defined as an effect, consequence, result, or repercussion created by the health technology on the characteristics and functioning of an organization or a set of organizations involved in the care or life pathway of users. To this end, the OIC helps to identify and structure the OIs according to the perspective of different actors, with a classification on the basis of three macrocriteria. To date, no information on how this institutional tool is used in health technology assessments has been published.

Based on the OIC consecutive steps published by the HAS, the objectives of this study were to use the OIC to assess in a structured manner the OI and to apply it on ICIs used for the management of advanced cancers have had on the health care system, on health care professionals (HCPs) and patients since their introduction based on literature review and interviews.

## METHODS

### Organizational Impact Cartography—Description

**FIG 1:**
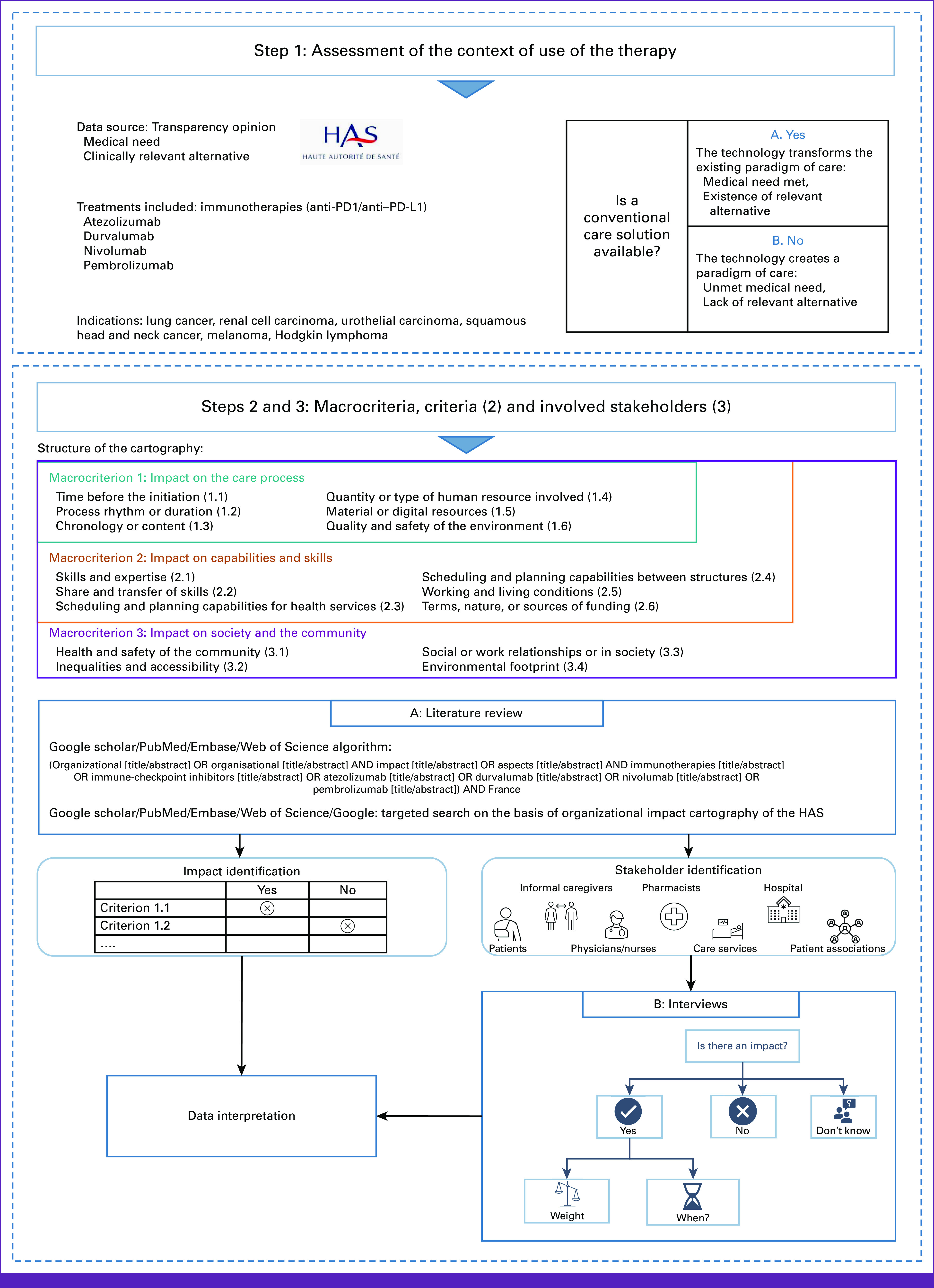
Organizational impact assessment process.

According to the HAS, an OI is evaluated through three consecutive steps: the global context of the assessment, the description of the OI using three macrocriteria and 16 subcriteria included in the cartography, and the identification of the actors concerned. The process used to assess the OI of immunotherapies is given in detail in Figure 1.

The first step aims to describe the global context of the technology under assessment. This part documents if the health technology evaluated either creates a new paradigm or modifies medical care as clinically relevant alternatives already exist.

The second part of the OIC is the classification of each possible impact according to the subcriteria and macrocriteria. The cartography is constituted of the following three macrocriteria: (1) impact on the care process, (2) impact on the capabilities and skills required of HCPs and patients to implement the care process, and (3) impact on the society and the community. Within each of these macrocriteria, four to six subcriteria are defined (Fig 1—step 2).

Regarding macrocriterion 1 relative to the care process, the OIC identifies six subcriteria exploring the impact on process evolution, the quantity or type of staff involved, the material or digital resources, and the quality and safety of the environment where the process takes place.

For the evaluation of the impact on the capabilities and skills required of HCPs and patients to implement the care process (macrocriterion 2), there are six criteria related to skills and knowledge, scheduling, working and living conditions, and funding.

Finally, for macrocriterion 3 relative to the societal and community impact, the OIC evaluates whether the technology might have an impact on the community in terms of health and safety, on social inequalities or accessibility of care, on social or work relationships in terms of society as a whole, and on the environmental footprint.

The third step aims to specify which stakeholders (if any) are concerned by an IO.

### Step 1: Global Assessment of the Context of the Use of Immunotherapies

To document this part, a review of all TC opinions of immunotherapies, which are indicated in advanced cancers in France (atezolizumab, durvalumab, nivolumab, and pembrolizumab, in monotherapy or in combination), was performed from 2014 (first ICI availability) until July 2021 in the following indications: Hodgkin lymphoma (HL), melanoma in adjuvant and metastatic settings, non–small-cell lung cancer (NSCLC), renal cell carcinoma, small-cell lung cancer, squamous cell carcinoma of head and neck, and urothelial carcinoma. On the basis of the medical need and the presence of relevant alternatives for each indication, the context is classified into one of two categories: (1) the immunotherapy changes the paradigm of care or (2) the immunotherapy creates a paradigm of care (Fig 1—step 1).

### Steps 2 and 3: Macrocriteria and Subcriteria to Assess the OI and Involved Stakeholders

#### Literature Review

For the scientific literature, PubMed database and conference proceedings for European Societies for Medical Oncology and French guidelines are searched. For other information, we perform searches on the Google platform and on websites of French regional and national health authorities, patients' associations, and hospitals. Newspaper articles and French government publications are reviewed.

In the targeted literature review, all publications presenting at least one organizational change listed in the OIC are retrieved. All descriptions of impact are then classified in the relevant criterion.

#### Interviews

Through the literature review, we identify the categories of actors potentially affected by the introduction of ICIs. Authors publishing on organizational change since the introduction of ICIs, ICI pathway or outcomes, and patients' association representatives are contacted by e-mail to propose a semistructured interview. Interviewees are asked whether someone else could be interested in the project or who should be interviewed (Fig 1—step 2). A target of at least 25-30 interviews is set to be representative of French metropolitan areas and HCPs and patients, with an opportunity to stop when information saturation is reached.

During the interviews, each person is asked if ICIs had an impact on each of the criteria included in the OIC. If they declare an impact, they must specify the period of the impact (ie, during the treatment introduction period [learning phase] or routine practice or both) and rank the impact as minor, moderate, and major. They have the opportunity to give their perspective and, for HCPs, the perspective of their patients (Fig 1—step 2).

### Data and Statistical Analyses

For the literature review, an impact on a criterion is defined when at least one publication mentioned it.

For the interviews, a quantitative analysis is first performed to estimate the percentage of HCPs and patients interviewed mentioning an impact. A consensus on impact is defined when at least 75% of the HCPs and patients mentioned an impact for any individual criterion.^8-10^ Second, the impact is weighted as follows: minor impact = 1, moderate impact = 2, and major impact = 4. An overall mean weight for each criterion is then calculated. The overall impact is also compared between categories of stakeholders (eg, physicians *v* nonphysicians). In the absence of a robust method to quantify a significant difference, a difference in impact count or weight between HCP categories of ≥30% is considered relevant. The second quantitative analysis focused on the timeframe of the impact to determine whether the impact occurs during the learning phase or in routine practice. The consensus to choose the period is fixed when at least 75% mentioned an impact.^8-10^

Using the literature and interviews, a qualitative analysis of the mentioned impact is performed.

## RESULTS

### Step 1: Assessment of the Context

The results of the 24 TC opinions are summarized in Table 1. For all opinions, clinically relevant alternatives already existed before the introduction of the OIC, except for durvalumab. On the basis of medical need defined as poorly or not covered, ICIs have created a new standard of care in four cases: durvalumab in NSCLC, nivolumab in adjuvant melanoma and HL, and pembrolizumab in adjuvant melanoma. For all other opinions, ICIs have transformed the existing paradigm of care.

**TABLE 1:**
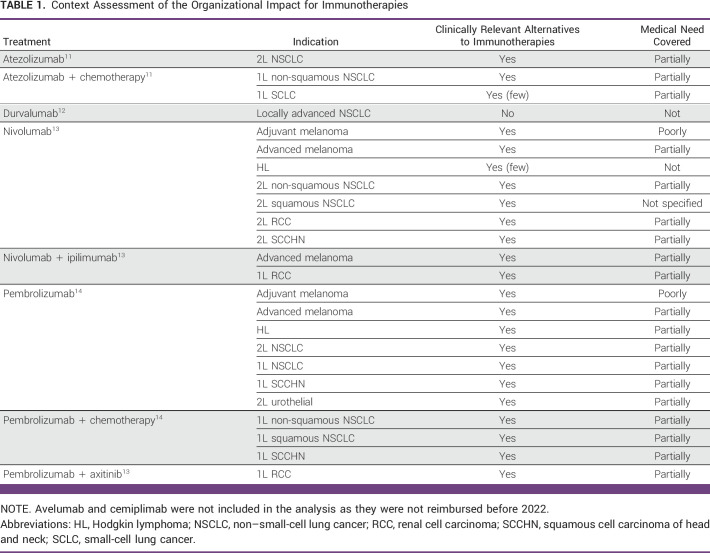
Context Assessment of the Organizational Impact for Immunotherapies

### Steps 2 and 3: Macrocriteria, Subcriteria, and Stakeholders

#### Literature Review

The literature review identified 58 publications or articles focusing on a potential OI according to the OIC criteria (Appendix Table A1). Impacts were observed for all three macrocriteria, concerning 11 of the 16 subcriteria. Publications explicitly mentioned no impact of the ICIs on one criterion (environmental footprint).^15-17^ No publication was identified for four criteria (1.1, 1.5, 1.6, and 3.1). HCPs publishing on OI or mentioned in publications were physicians (oncologists, specialists, immunologists, and general practitioners), pharmacists, nurses, and patient associations.

#### Interviews

Overall, 27 interviews were conducted. HCPs and patients' association representative were from most French regions and mostly women (56%). Most stakeholders were physicians (56%), specializing in oncology (n = 6), lung medicine (n = 2), urology or nephrology (n = 1), dermatology (n = 2), and immunology (n = 3), and one general practitioner. Other stakeholders were pharmacists (n = 6), nurses (n = 3), or patient association representatives (n = 3). HCPs included in the study worked in hospital, home care facilities, regional health care institutions, or academic societies.

There was a consensus on impact between the interviewees for a quarter of the criteria (n = 6 of 16). A quarter of the criteria have had a high impact according to weighting (n = 6 of 16).

**FIG 2:**
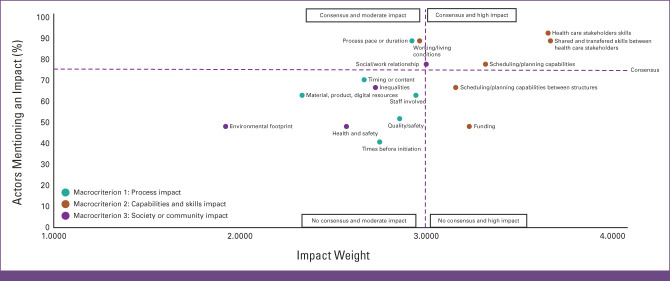
Percentage and weight of impacts. Seventy-five percent of actors mentioning an impact corresponds to consensus. Impact weight of ≥3 corresponds to high impact.

Four criteria (health care stakeholders' skills [2.1], transferred skills between health care stakeholders [2.2], scheduling capabilities [2.3], and social relationship [3.3]) achieved high consensus and were rated as having a high impact; two criteria (rhythm or care duration [1.2], working/living conditions [2.5]) achieved high consensus with a moderate impact. Two criteria (funding and scheduling capabilities cross-structure) were considered to have a high impact, but no consensus was achieved. For eight criteria, there was no consensus and impact was rated as moderate (Fig 2). A consensus of impact occurring in both periods was observed for half the criteria (Appendix Table A2).

**FIG 3:**
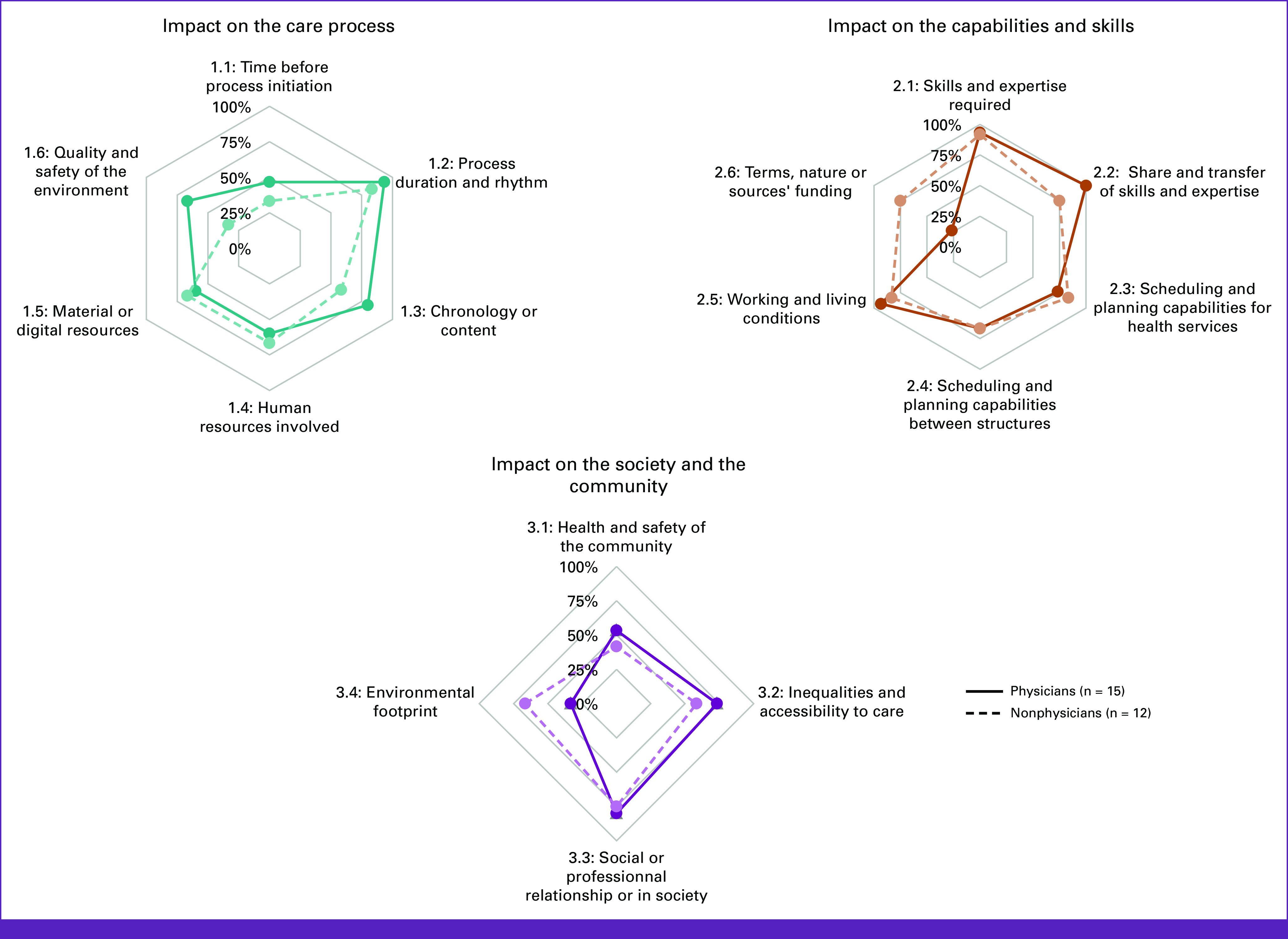
Percentage of impact per health care stakeholders' category.

Stakeholders' categories perception differed for three criteria. Most physicians considered that there was an OI on the quality and safety of the environment (criterion 1.3) and on the context in which the process takes place (criterion 1.6), whereas this was not the case for nonphysician stakeholders (Fig 3). On the contrary, most nonphysician stakeholders more frequently identified an OI on the terms, nature, or source of stakeholder funding and on the environmental footprint.

### Comparison Between the Literature Review and the Interviews

The interviews enabled us to identify OI for more criteria than the literature review. The main differences concerned the impact on the care process.

Regarding the criteria with a high impact and/or consensus (Table 2), interviews enabled us to capture more information on the OI of ICIs except for the scheduling and planning capacities for health care services (2.3). Both sources agreed on the impact on scheduling and planning because of increased treatment duration (until disease progression or with a stopping rule; 1.2) and service saturation (1-day admission and pharmacy production; 2.3). Interviews mentioned the impact on resources in pharmacy services (2.3) caused by increased patient flow and incubator saturation. On the other hand, the literature review identified the difference in capabilities between hospital center to cope with saturation. The adverse events caused by immunotherapies also affected the resources needed. For example, interviews mentioned the increased duration of pharmacovigilance dossier management and difficulty in predicting the treatment duration of ICIs (1.2). Moreover, the transfer of patients between hospitals for treatment or adverse events management (2.4) was identified in both sources. In interviews, HCPs also mentioned the impact for patients since they are principally followed by specialists in university hospitals, which may differ from the hospital where their treatment is administered. The introduction of ICIs has affected the knowledge and expertise of the HCPs. Indeed, both sources mentioned the need for training of HCPs and patients caused by new types of adverse events (2.1) and the transfer of knowledge between hospital HCPs (2.2).

**TABLE 2:**
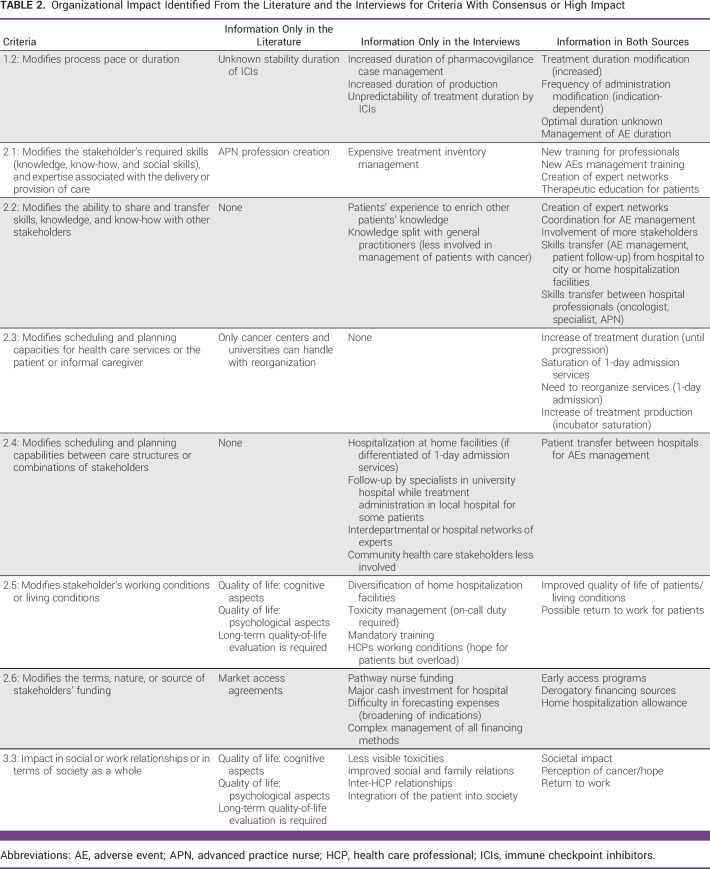
Organizational Impact Identified From the Literature and the Interviews for Criteria With Consensus or High Impact

## DISCUSSION

To our knowledge, this study provides for the first time an assessment of the OI of immunotherapies on the basis of the cartography developed by the HAS. Immunotherapies have transformed patient management in most cases.

Immunotherapies have had a strong OI, especially on the criteria related to the capabilities and skills required to implement the care process, all of which achieved consensus or were defined as high impact. Given the increased number of ICI indications, their effectiveness at prolonging survival, and the increased treatment duration until progression (or according to a stopping rule), the number of patients treated with ICI has increased over time.^18,19^ In France, between 2019 and 2020, the number of patients treated with ICI increased by more than 40%.^19^ The increased patients flow has had a major impact on the volume of treatments to be produced in hospital pharmacies and led to a saturation of outpatient hospitalization services. Consequently, some hospital centers have implemented a monitoring program to optimize treatment production and organization of outpatient services. In parallel, experimentations with dose and schedule optimization and home treatment administration have been implemented.^20-22^

ICIs are associated with specific immune-related side effects.^23^ More than half of patients treated will experience an acute or delayed adverse event (AE), sometimes chronically (up to 40%).^24-27^ Given the nature of AEs associated with ICIs, oncologists and specialists have needed further training to liaise with immunologists to manage these AEs appropriately. HCPs have had to create expert networks rapidly to share knowledge affecting collaboration between care structures or services (2.4). In France, there are at least 16 multidisciplinary consultation meetings and 25 expert networks for AE management.^28^ The outcome of these meetings should be shared widely to implement current guidelines.^29,30^

Apart from the impact on duration (1.2) and relationships (3.3), for the criteria related to the process of care (MC1) and to societal impact (MC3), no consensus was obtained and their impact was considered mild or moderate. The significant impact on capacity and skills might be explained by the panel interviewed. Certain criteria such as social inequalities (3.2)^31^ or the environmental footprint (3.4)^32^ may merit expert evaluation by specialists in these domains.

The main limitation of this study is related to knowledge of the OIC. Until the time of writing, the HAS has not published any guidelines regarding the method for criteria selection and how the results should be exploited, questioning the acceptability by the HAS of the methodology reported in this study. Moreover, the representativeness of each stakeholder category is questionable. However, physicians (almost half the sample) are the professionals most frequently dealing with immunotherapy and our findings were in general comparable between physicians and nonphysicians for 13 criteria. Therefore, for certain criteria such as funding, social inequalities, or the environmental footprint, relevant expert opinion should be considered in further analyses. Furthermore, HCPs did not have a thorough knowledge of the OIC in terms of composition and interpretation of the criteria but were interested to participate in the study that had probably biased or affected their answers. Although the HAS guidelines were designed to assess the OI of individual drugs or health technologies in each indication or setting, we explored the OI of a therapeutic class across all indications. As a result, our study did not capture any specificities that may exist within individual immunotherapies or individual indications. However, evaluating a therapeutic class as a whole also provides relevant information. For example, the expansion of indications for ICIs is the reason why the saturation of 1-day admission capacity is highlighted (criterion 2.3). We believe that evaluation of OI by therapeutic class should also be considered by the health authorities to have a comprehensive view of the impact of drug access. To our knowledge, this is the first study exploring how to conduct an OI impact assessment on the basis of the OIC prepared by the French HAS. In addition to a targeted literature review, semistructured interviews with 27 health care stakeholders enabled a broad spectrum of the different facets of patient management to be explored.

OI assessments are of growing importance for HTA, and both European Network^33^ and the National Institute for Health and Care Excellence have shown interest in the question. In particular, the scale of OI assessments needs to be discussed. However, organization systems are specific for each country, and even within individual countries, there are multiple differences between hospitals. Questions remain on how the assessment of OI should be made. This study showed that a consensus on the impact of a criterion is not the only feature that should be considered important or relevant but that the magnitude of the impact also needs to be taken into account. Moreover, assessment of the OI of individual drugs in individual indications may underestimate a major OI caused by the multiplication of indications for certain drugs.

In conclusion, a literature review and interviews should be considered for OI assessment. The introduction of ICI has had a major OI in France, especially regarding capacities and skills of health care stakeholders. For hospital outpatient services, we recommend an assessment of incoming patient flow and of the treatment duration of ICI by indication to estimate the required resources better. To prevent saturation of outpatient services, dose or schedule optimization and home treatment should be more widely considered. Finally, to enrich knowledge on AEs, the findings of expert networks should be more widely shared.

## Figures and Tables

**TABLE A1:**
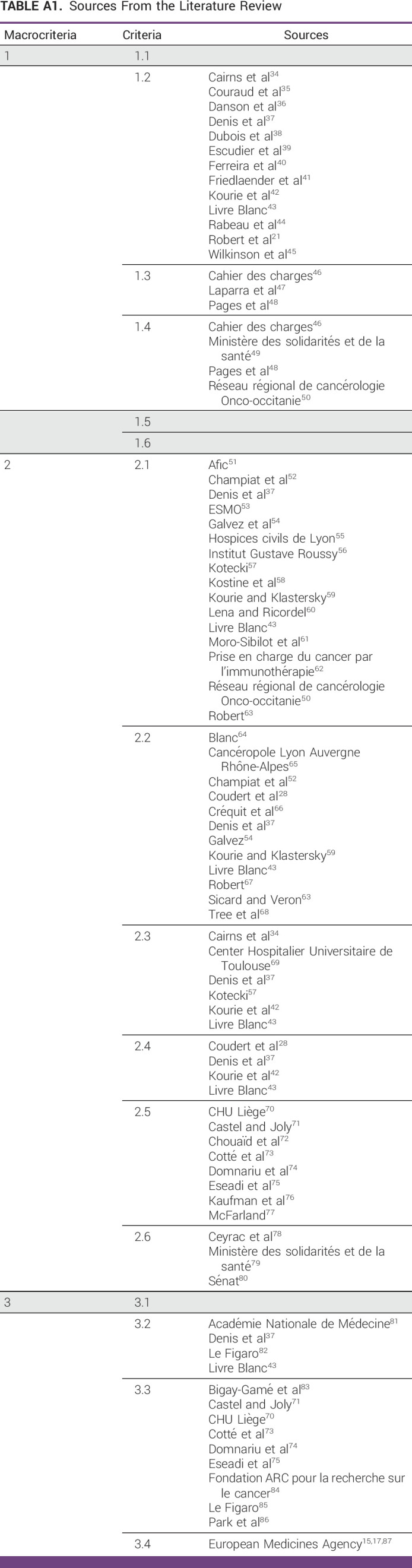
Sources From the Literature Review

**TABLE A2:**
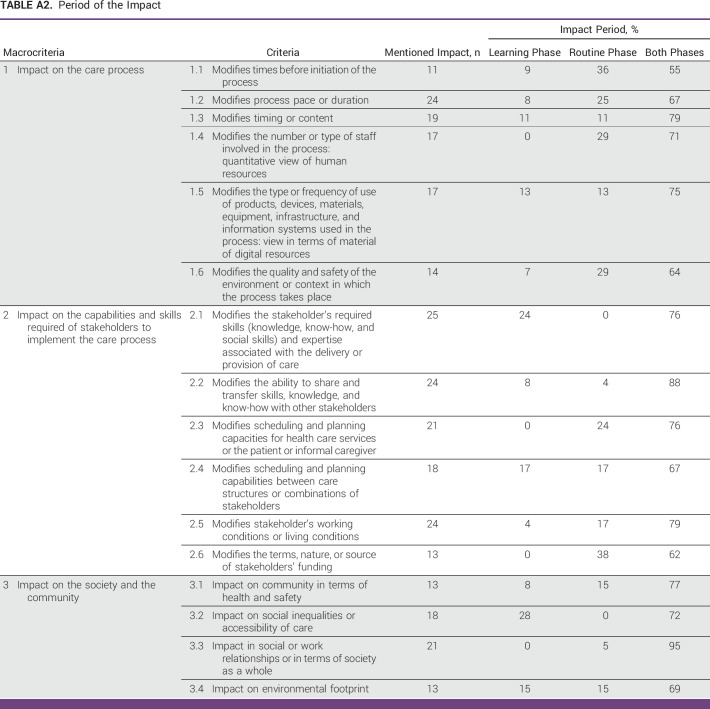
Period of the Impact
